# Smoking cessation among diabetes patients: results of a pilot randomized controlled trial in Kerala, India

**DOI:** 10.1186/1471-2458-13-47

**Published:** 2013-01-18

**Authors:** KR Thankappan, GK Mini, Meena Daivadanam, G Vijayakumar, PS Sarma, Mark Nichter

**Affiliations:** 1 Achutha Menon Centre for Health Science Studies, Sree Chitra Tirunal Institute for Medical Sciences and Technology, Trivandrum, Kerala, 695 011, India; 2 Quit Tobacco India Project, Achutha Menon Centre for Health Science Studies, Sree Chitra Tirunal Institute for Medical Sciences and Technology, Trivandrum, Kerala, 695 011, India; 3 Medical Trust Hospital, Pandalam, Pathanamthitta, Kerala, India; 4 Family Medicine and Public Health, University of Arizona, Tucson, Arizona, USA

**Keywords:** Diabetes, Smoking cessation, Counseling, Kerala, India

## Abstract

**Background:**

India has the second largest diabetic population (61 million) and tobacco users (275 million) in the world. Data on smoking cessation among diabetic patients are limited in low and middle income countries. The objective of the study was to document the effectiveness of diabetic specific smoking cessation counseling by a non-doctor health professional in addition to a cessation advice to quit, delivered by doctors.

**Methods:**

In our parallel-group randomized controlled trial, we selected 224 adult diabetes patients aged 18 years or older who smoked in the last month, from two diabetes clinics in South India. Using a computer generated random sequence with block size four; the patients were randomized equally into intervention-1 and intervention-2 groups. Patients in both groups were asked and advised to quit smoking by a doctor and distributed diabetes specific education materials. The intervention-2 group received an additional diabetes specific 30 minutes counseling session using the 5As (Ask, Advise, Assess, Assist and Arrange), and 5 Rs (Relevance, Risks, Rewards, Roadblocks and Repetition) from a non-doctor health professional. Follow up data were available for 87.5% of patients at six months. The Quit Tobacco International Project is supported by a grant from the Fogarty International Centre of the US National Institutes of Health (RO1TW005969-01).

The primary outcomes were quit rate (seven day smoking abstinence) and harm reduction (reduction of the number of cigarettes / *bidis* smoked per day > 50% of baseline use) at six months.

**Results:**

In the intention to treat analysis, the odds for quitting was 8.4 [95% confidence interval (CI): 4.1-17.1] for intervention-2 group compared to intervention-1 group. Even among high level smokers the odds of quitting was similar. The odds of harm reduction was 1.9 (CI: 0.8-4.1) for intervention-2 group compared to intervention-1 group.

**Conclusions:**

The value addition of culturally sensitive diabetic specific cessation counseling sessions delivered by non-doctor health professional was an impressive and efficacious way of preventing smoking related diabetic complications.

**Trial Registration:**

Clinical Trial Registry of India (CTRI/2012/01/002327)

## Background

India has the second largest population (1210 million) [1] and number of people with diabetes (61 million) [2] and tobacco users (275 million) [3] after China. Both diabetes prevalence and tobacco use are increasing rapidly in India. Although the proportion of smokers among all types of tobacco users in India was only 40.5% in 2010, in contrast to other countries, smoking was predicted to cause about one million deaths [4]. Smoking is strongly linked to the risk of diabetes morbidity as well as mortality [5-7]. The International Diabetes Federation in 2003 [8] and the American Diabetes Association in 2004 [9] have both strongly recommended that people with diabetes not to smoke because of increased risk of diabetes complications. The major complications are cardiovascular diseases [10], stroke [11], diabetic retinopathy [12], and peripheral arterial disease [13].

Kerala, the Indian state most advanced in epidemiological transition [14] and the state with the highest prevalence of diabetes, was reported as the harbinger of what is going to happen to the rest of India in the near future [15,16]. Current smoking prevalence of 27.9% among Kerala men was higher than the 24.3% for the whole of India [3]. A previous Quit Tobacco International (QTI) study in Kerala found that 59% of diabetes patients were tobacco users (43.5% exclusive smokers) prior to diagnosis and more than half of these users continued to use tobacco, many daily, even after diagnosis. Notably, 52% had not been advised to quit smoking by their doctors and did not associate smoking with diabetes complications [17]. Given the prevalence of smoking among diabetics, there was clearly a need for proactive cessation efforts. Results of a randomized controlled trial from the US found that smoking cessation intervention using motivational interviewing integrated into an established diabetes self management training program curriculum resulted in a trend towards greater abstinence at three months of follow-up in those receiving the directed smoking cessation intervention [18]. Data on smoking cessation among diabetic patients are limited in low and middle income countries. An Indonesian study of 71 diabetic patients demonstrated the feasibility of disease-centred doctors’ messages about smoking cessation for these patients in a clinic setting [19]. Considering the limited access to doctors in India, particularly in rural areas, [20] there is a need to utilize the services of non-doctor health professionals for smoking cessation more frequently than doctors. The objective of the study was to document the effectiveness of diabetic specific cessation counseling by a non-doctor health professional in addition to a diabetic specific cessation message to quit, delivered by doctors.

## Methods

### Participants

**Figure 1:**
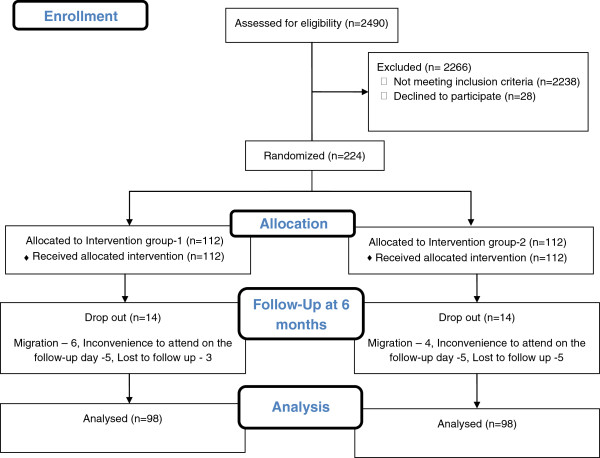
Patient flow diagram.

All of the 2490 male diabetic patients (aged 18 years and above) who attended two referral diabetes clinics located in peri-urban areas of two south Indian cities located in Kerala state were screened for smoking through the use of a brief instrument provided to patients at the clinic registration counter from December 2008 to April 2011. Among them 14.6% (n=363) were current smokers. Being a pilot study we decided to include all the patients who satisfied the inclusion criteria during the recruitment period. Inclusion criteria for the study were: male diabetes patients aged 18 years or above, literate, native to the clinic catchment area, high probability that they would be treated at the clinic for the next six months and willingness to participate in the study. After excluding patients who did not meet inclusion criteria, or did not agree to participate in the study (n=139) a total of 224 male diabetes patients (mean age 53 years) who smoked in the previous month were selected for the study. Female patients were excluded since the smoking rate among females in Kerala was zero percent [3]. Ethical clearance for the study was given by the Sree Chitra Tirunal Institute for Medical Sciences and Technology, Trivandrum. Written consent to participate was obtained after patients were informed about the purpose of the study. Patients were a mix of newly diagnosed and long time patients (Figure 1).

### Study procedure

The procedure followed was minimally invasive. A screening instrument was kept at the hospital reception where all the patients had to register after entering the hospital. Smokers were identified by the counselor from this screening tool which inquired the patient’s smoking status. Patients attending the clinic routinely go to the lab for a blood glucose examination and then have a waiting period before lab results are ready and they can be seen by the doctor. During this time, the counselor met patients who had indicated a history of smoking on the screening instrument. After being informed about the study details written consent from the patients was obtained. From those who gave consent, the counselor collected baseline information using a pre tested structured interview schedule. Details about basic demographic information, smoking history, current smoking patterns and presence of any other chronic diseases (hypertension, cardiovascular disease, stroke, chronic obstructive pulmonary disease, and cancer) were collected.

Subsequently the counselor randomized the patients equally into two groups; intervention–1 and intervention–2 groups, with block size four. Sequentially, every four patients enrolled were randomized into the two intervention groups using a computer generated random sequence to achieve a block size of four, to facilitate interim analysis. Their medical records were then flagged with different colored stickers by the counselor in order to identify group assignment. After the interview, the patient consulted the doctor. The doctor gave the patient a standard diabetes specific tobacco cessation message. The doctor also showed the patient visual images of common diabetes complications exacerbated by smoking. At the end of consultation the doctor instructed the patient to meet with the counselor. The counselor provided educational materials on tobacco and diabetes developed by the QTI on the harm of tobacco for diabetes patients that built on formative research and followed a question answer format for all the patients [21] and gave follow up dates for consultation to all patients.

Intervention–2 group patients received three diabetic specific tobacco counseling sessions (at first contact, at one month and at three months) lasting about 30 minutes in each session following the 5 ‘A’s (Ask, Advise, Assess, Assist and Arrange) and 5 ‘R’s (Relevance, Risks, Rewards, Roadblocks and Repetition) [22]. In this session, after going over the educational material, developed by the QTI for smoking cessation, with the patient (to establish relevance and support the doctor’s advice) the counselor assessed each patient’s readiness to quit. If ready to quit, the counselor assisted him by discussing practical quit tips, how to get through an initial period of withdrawal, and how to deal with common withdrawal symptoms, emphasizing that these only lasted for a few days. If not ready to quit, the counselor briefly identified roadblocks and challenges to quitting, and encouraged the patient to think about quitting after reconsidering the risks of smoking for developing diabetes complications and the benefits of quitting as a means of preventing complications as a prime motivator.

All patients were given smoking cessation advice on each visit by the doctor for the next six months. Participants in intervention–2 group additionally received face to face counseling sessions on each visit for the next six months. Thus patients in the intervention group-2 received three counseling sessions: first at baseline, second at month one and third at month three of follow-up.

### Training

The doctors and diabetes educators selected to counsel patients in the study sites were initially given training on the harm of tobacco for diabetes patients including: 1) a review of epidemiological data on smoking as a diabetes risk factor, 2) complications strongly associated with smoking among those afflicted with diabetics, and 3) the mechanisms through which smoking contributes to vascular constriction and obstructed blood flow. Educational materials developed by the QTI for diabetes patients that explain these facts in simple terms were provided to the counselor to give to patients. Doctors and the counselors were also trained in basic brief intervention cessations skills. Doctors were instructed to ask all patients about their smoking status and to strongly advise patients not to smoke using a standardized diabetes specific cessation message linking smoking to the complications of diabetes. Doctors were provided a visual aid illustrating how tobacco narrows the passage of blood in the vascular system and pictures illustrating diabetes complications at distal points of the vascular system such as eyes, feet, fingers, and penis. In sum, the doctors were instructed to actively deliver two of the Five As, Ask and Advise, using illness specific visual aids. The counselors were given additional training in tobacco cessation counseling and instructed to actively conduct all of the five ‘A’s with patients in intervention group–2 each time they attended the clinic and 5 ‘R’s when necessary. The counselors were instructed to document the details of cessation offered to at least 15 patients using five ‘A’s and five ‘R’s. An examination was conducted by one of the authors (MN) based on these 15 brief interventions to assess their cessation skills. A certificate titled “basic tobacco cessation competency” was issued on successful performance in the examination by the University of Arizona.

### Follow-up of patients

Follow up interviews were conducted with all study participants in both groups at one month, three months and six months. All the follow-up interviews were conducted in person, although some of them were reminded by phone calls to come for the follow-up visit. Patients were asked about their smoking in the last seven days as well as questions related to the number of cigarettes/*bidis* used in an average day. We reminded all the patients in both the groups by phone about the six month follow up in order to get maximum response. All the diabetic patients followed up at one, three and six months were seen by the doctor and were advised to quit smoking.

### Outcome measure

The primary outcome measure was a seven day smoking abstinence (quit rate) measured by a question: “During the past seven days, did you smoke even a puff?” Other smoking outcomes gathered included patients’ reports on the number of cigarettes/*bidis* smoked on an average day of use.

### Statistical analysis

Statistical comparisons of means and proportions were made using Student’s t-tests, Chi-square tests, Chi-square tests for trend or Fisher’s exact test. The relative risk was estimated by computing odds ratios (OR). Multivariable models using multiple logistic regression analyses were used to identify the correlates of quit rates. A complete case analysis and intention to treat analysis were done. In order to test our hypothesis that high level smokers are more addicted to smoking and less likely to quit smoking compared to their low level smoking counterparts, we did a stratified analysis of baseline level smoking and quit rate at six months. All the analyses were done using SPSS version 17.0 and statistical significance was set at two tailed p<0.05. The statistician was blinded to group assignment.

## Results

We screened 2490 male diabetic patients. Among them 363 (14.6%) were current smokers. Of these 363 patients, after excluding patients who were not willing to participate in the study (n=31), who were not the natives to the clinic catchment area (n=89) and who could not come for follow ups for the next six months (n=19), a total of 224 patients were included in the final study.

Average age of the study patients was 53 years (range 28–75). Under the age of 40 years there were 17 patients (7.6%), confirming previous findings of the early onset of diabetes in India [23]. Median duration of diabetes was six years compared to the mean duration of eight years. Close to three-fourths of the patients were subjectively assessed by the counselors as belonging to the middle socioeconomic status (SES) group, which was similar to the SES of the general population in Kerala [24]. Baseline characteristics in both the intervention groups were comparable (Table 1).

**Table 1:** Baseline characteristics

**Background characteristics**	**Intervention group-1**	**Intervention group-2**	**P value**
	**N=112**	**N=112**	
Mean age (years) ± SD	54.2 ± 8.8	52.5 ± 9.9	0.193
Mean age of initiation of smoking (years) ± SD	20.9 ± 8.1	21.2 ± 5.6	0.723
Mean age at diagnosis of diabetes (years) ± SD	46.3 ± 9.2	44.5 ± 10.7	0.193
Mean duration of diabetes (years) ± SD	7.9 ± 6.1	8.0 ± 6.6	0.897
Mean number of sticks used per day at baseline ± SD	15.0 ± 14.6	14.1 ± 13.2	0.640
Currently Married (%)	98.2	94.6	0.140
Others (%)	01.8	05.4	
< 10 years of schooling (%)	27.7	20.5	0.137
≥ 10 years of schooling (%)	72.3	79.5	
Working (%)	61.6	66.1	0.289
Not working (%)	38.4	33.9	
Low SES (%)	24.1	17.9	
Middle SES (%)	72.3	75.9	
Upper Middle SES (%)	03.6	06.3	0.376
Presence of any other chronic diseases (%)	39.3	38.4	0.500

SD = Standard Deviation. SES= Socioeconomic Status.

The mean age of initiation of smoking was 21 years (SD 6.9, range: 8–56 years). Around 44% of patients initiated smoking in their adolescent years (< 20 years). Twenty eight patients (12.5%) were diagnosed in the last two years prior to the study. Mean age of diagnosis of diabetes was 45.4 years (SD: 10.1, range: 22–71 years). Thus on an average these patients smoked 24 years before the diagnosis of diabetes.

In the first follow-up wave (month one) we were able to contact 173 (77.2%) patients, in the second follow-up (month three) 163 (72.8%) patients and in the third follow-up (month six) 196 (87.5%) patients.

### Quit rate and harm reduction

Smoking status of the patients at the six-month follow up based on complete case analysis is given in Table 2 and that based on intention to treat analysis in Table 3.

**Table 2:** Smoking status at six months follow up using complete case analysis

**Outcome**	**Intervention group–1 (n=98)**	**Intervention group–2 (n=98)**	**Unadjusted OR (95% CI)**	Adjusted OR* (95% CI)	**p value for adjusted OR**
	**n (%)**	**n (%)**			
Quit rate	14 (14.3)	58 (59.2)	8.7 (4.3-17.4)	10.7 (5.1-22.7)	<0.001
Harm reduction	25 (29.8)	20 (50.0)	2.3 (1.1-5.1)	2.6 (1.1-5.8)	0.025

*Adjusted for age, education, occupation, presence of any other chronic disease, duration of diabetes, volume of counseling sessions received and number of sticks per day. Quit Rate**=** Point prevalence abstinence of no smoking in the last seven days. Harm reduction=Reduction of smoking (number of sticks per day) more than 50% of baseline use. OR=Odds Ratio. CI = Confidence Interval.

**Table 3:** Smoking status at six months follow up using intention to treat analysis

**Outcome**	**Intervention group-1 (n=112)**	**Intervention group-2 (n=112)**	**Unadjusted OR (95% CI)**	Adjusted OR* (95% CI)	**p value for adjusted OR**
	**n (%)**	**n (%)**			
Quit rate	14 (12.5)	58 (51.8)	7.5(3.8-14.7)	8.4 (4.1-17.1)	<0.001
Harm reduction	25 (25.5)	20 (37.0)	1.71(0.84-3.5)	1.9 (0.8-4.1)	0.101

*Adjusted for age, education, occupation, presence of any other chronic disease, duration of diabetes, volume of counseling sessions received and number of sticks per day. Quit Rate**=** Point prevalence abstinence of no smoking in the last seven days. Harm reduction=Reduction of smoking (number of sticks per day) more than 50% of baseline use OR=Odds Ratio. CI = Confidence Interval.

Data from the six-month follow-up was available for 196 patients (87.5%). The odds for quitting was 10 times higher for intervention–2 group compared to intervention–1 group in the complete case analysis and close to nine times higher in the intention to treat analysis. Harm reduction (defined as > 50% reduction in the number of cigarettes/*bidis* used per day compared to baseline use), which was significantly higher in the intervention–2 group, was not found to be significant in the intention to treat analysis. The mean number of cigarettes/*bidis* smoked per day at month six was 4 (SD 8.2) in the intervention–2 group, significantly lower (p < 0.001) than that of 10 (SD 13.7) in the intervention–1 group in complete case analysis.

The quit rate based on intention to treat analysis at the one-month follow-up between the intervention–1 group (11.6%) and the intervention–2 group (19.6%) was not statistically significant (p = 0.09). At three months follow up the quit rate remained at almost the same level in the intervention–1 group (10.7%), whereas in the intervention–2 group the quit rate increased to 28.6% and the difference was statistically significant ( p <0.001). At the six-month follow-up the quit rate further increased to 51.8% in the intervention group–2. Among those who came for all the three follow up visits in this group, statistically significant (p=0.007) positive trend in quit rate was seen with increase in the number of counseling sessions attended.

Readiness to quit was assessed only for intervention group-2 as part of the intervention strategy. Out of the 112 patients in the intervention – 2 groups 77 reported that they were ready to quit at baseline. At six month follow-up, 40 patients (51.9%) out of these 77, quit smoking where as among the 35 patients who were not ready to quit at baseline, 18 (51.4%) quit smoking at six month follow-up.

Quit Rate at six months by baseline level of smoking is given in Table 4. Although the quit rates among low and high level smokers significantly increased in the intervention-2 group, the increase in quit rate among the medium level smokers did not achieve statistical significance probably due to small sample size. However, 72% of the medium level smokers shifted to low level smoking at the end of six months.

**Table 4:** Quit Rate at six months by baseline level of smoking: Intention to treat analysis results

**Baseline level of smoking**	**Quit rate**		**P value**
	**Intervention group-1**	**Intervention group-2**	
	**N (%)**	**N (%)**	
Low ^1^	5/32 (15.6)	25/39 (64.1)	< 0.001
Medium^2^	4/30 (13.3)	8/24 (33.3)	0.105
High^3^	5/50 (10.0)	25/49 (51.0)	< 0.001
Total	14/112 (12.5)	58/112 (51.8)	< 0.001

^1^Smoked 1–5 sticks (cigarettes / *bidis*) per day, ^2^ smoked 6–10 sticks per day, ^3^smoked more than 10 sticks per day.

## Discussion

The study found that both the doctor’s message alone and counseling lead many patients to quit or significantly reduce their smoking habit. This was true of both low level smokers at baseline and high level smokers. Quit rates in the intervention–1 group were 12.5% at month six compared to the baseline, indicating the importance of routine smoking cessation advice by doctors to all diabetes patients. Doctor’s cessation advice that is disease specific is responded to better by patients than general advise [21]. Our finding of a nearly nine times higher quit rate (seven day abstinence from smoking) of smoking in the intervention–2 group of diabetes patients compared to the intervention–1 group indicates that trained non-doctor health professional increases the chances a patient will quit significantly.

The only prior study from a developing country on cessation among diabetes patients from Indonesia reported a quit rate of 30% in the group that received doctor’s advice and 37% in the group that received doctor’s advice and counseling. Although this difference in quit rate was not statistically significant, the quit rate of both groups’ was significantly higher at the six-month follow-up compared to the base line. The quit rate of 52% in our intervention–2 group was much higher than the 37% in Indonesia. This could be due to several factors, including the lower average number of cigarettes/*bidis* smoked per day in Kerala compared to Indonesia, the highly educated population in Kerala, better implementation of the national tobacco control program and the repeated 30 minutes counseling sessions for quitting each time the patient visited the clinic. High quit rates of 50% at one year follow-up was reported by chronic obstructive pulmonary disease (COPD) patients in a recent study on smoking cessation buddies in COPD indicating that high quit rates are possible in chronic disease patients [25].

Generally, people including health professionals do not associate smoking with diabetes. Awareness of the association between smoking and cancer, cardiovascular diseases and respiratory diseases are generally higher [26]. However, it was reported in a previous study from Kerala that close to two thirds (64%) of diabetes patients reported that smoking will not affect the disease and only 10% reported that smoking causes a lot of aggravation of diabetes [27]. In our study both the doctor and the counselor used visual aids and diabetes specific smoking cessation materials developed by the QTI to motivate patients to consider quitting to prevent complications from diabetes.

This study found a dose response relationship between counseling and quit rate. The quit rate increased significantly from the one-month follow-up to the third month and again at the six-month follow-up. This demonstrates the significance of repeat counseling at frequent intervals for increasing quit rates and probably sustaining it. It is important to treat smoking as a chronic disease understanding the nature of addiction, possibility of relapse and the need for continuum of care [28]. The doctor employed the 2As (ask and advise) in their brief intervention lasting three minutes. Non-doctor health professionals who were trained as cessation counselors employed all five ‘A’s and 5 ‘R’s adapted to the Indian context during their 30 minute counseling sessions. Their assisting patients to recognize the risks of smoking and benefits of quitting, and to face physical, psychological and social roadblocks to quitting and plan quits resulted in higher quit rates over time.

The Indian Institute of Diabetes in Kerala, one of our study sites for this study, has taken note of the outcome of this study and is currently planning to incorporate smoking cessation counseling as a routine activity in their diabetic clinics and advise the State Government to follow this practice.

### Limitations of the study

Our study followed the patients only for six months, the outcomes were self reported and we did not conduct biochemical verification due to resource constraints. However, it has been reported by the Society for Research on Nicotine and Tobacco subcommittee on biochemical verification that for population based trials with low demand situation biochemical verification may not be necessary [29]. The counselors who assessed the outcomes were not blinded to the allocation groups while collecting follow up data, although the statisticians who analyzed the data were blinded. Since zero percent of women in Kerala smoked they were excluded from this study [3]. With a large proportion of diabetic smokers seemingly ineligible for the study, another limitation is that the results may apply to only specific smokers (male, literate, clinical care at the same site over six months).

## Conclusion

All doctors should routinely ask and advise diabetes patients to quit smoking, including calling attention to the complications that are more likely if they continue to smoke. We have demonstrated that a brief intervention by doctors is likely to result in a quit rate of about 10–13%. If this brief intervention is further supported by counseling sessions by a trained non-doctor health professional, the patients were able to reach a 52% quit rate at the six-month follow-up. Even among the high level smokers the quit rate at six months was 51%. And of those who did not quit, at least 25% engaged in harm reduction by reducing their former smoking levels to more than 50% of baseline. Counseling sessions using disease specific diabetes messages and culturally sensitive use of the five ‘A’s and five ‘R’s cessation protocol is an efficacious way of reducing smoking, an important risk factor that significantly increases the chances of life threatening, debilitating and costly diabetes complications.

If this system of brief intervention by the doctors supported by a counseling session by a non-doctor health professional can be incorporated in the national health system, a substantial proportion of diabetes complications due to smoking can be prevented.

## Competing interests

The authors declare that they have no competing interests.

## Authors’ contributions

The contribution of each of the authors is listed below: KRT: Conception, design, revision of the manuscript with critical contribution for intellectual content, and giving final approval of the version to be published, GKM: Conception, design, analysis and interpretation of data, and revision of the manuscript for intellectual content, MD: Conception, design, analysis and interpretation of data and drafting of the manuscript, GV: Conception, design, and revision of the manuscript with critical contribution for intellectual content, PSS: Analysis, interpretation of data, and contribution to critical revision of the manuscript for statistical accuracy, MN: Conception, design, analysis and interpretation of data, and revision of the manuscript for intellectual content. All authors read and approved the final manuscript.

## Pre-publication history

The pre-publication history for this paper can be accessed here:

http://www.biomedcentral.com/1471-2458/13/47/prepub
